# Rash decisions: Unmasking a risk phenotype in adults with persistent delayed penicillin allergy sensitized during historic infection with Epstein-Barr virus

**DOI:** 10.1016/j.jacig.2024.100320

**Published:** 2024-08-05

**Authors:** Fionnuala Cox, Natasha E. Holmes, Jamie Lee Waldron, Jason A. Trubiano

**Affiliations:** a Department of Infectious Diseases, Centre for Antibiotic Allergy and Research, Austin Health, Heidelberg, Victoria, Australia; b Department of Infectious Diseases, The Peter Doherty Institute for Infection and Immunity, University of Melbourne, Melbourne, Victoria, Australia; c Department of Medicine, Division of Allergy and Immunology, Massachusetts General Hospital, and Harvard Medical School, Boston, Mass

**Keywords:** Penicillin allergy, EBV, Maculopapular exanthema, DRESS, delayed drug rash, allergy testing

## Abstract

**Background:**

Penicillin-associated exanthems in the setting of infectious mononucleosis caused by Epstein-Barr virus (EBV) are often viewed as a transient event, not a true allergy. Recent evidence challenges this and suggests that a notable subset of patients retain penicillin hypersensitivity.

**Objective:**

We investigated the occurrence and predictors of persistent adulthood hypersensitivity in those with penicillin-associated rash occurring in the setting of EBV infection.

**Methods:**

Retrospective analysis of data of patients referred for penicillin allergy testing to an Australian tertiary-care hospital captured from 2015 to 2023 was carried out.

**Results:**

Of 2066 patients, 23 (1%) had penicillin-associated rash during an historic EBV infection; 16 (70%) were female; and median (interquartile range) age was 18 (16-20) years at index reaction and 38 (33.5-57) years at allergy testing. Skin prick testing and delayed intradermal testing to a penicillin panel were performed, followed by oral provocation challenge in those testing negative. Persistent sensitization was shown in 6 (26%) of 23; 4 (67%) of 6 positive delayed intradermal testing; and 3 (50%) of 6 had positive oral challenge test. Notably, 5 (83%) of 6 had a severe maculopapular exanthem with facial swelling, including 2 (33%) of 6 with probable drug reaction with eosinophilia and systemic symptoms (aka DRESS) during the index reaction, compared to 0 of 17 in patients tolerating penicillin on reexposure.

**Conclusion:**

This study highlights the requirement of allergy testing in adult patients reporting a penicillin-associated severe maculopapular exanthem in the setting of EBV, even if it occurred during childhood or adolescence.

Penicillin-associated exanthems in the setting of infectious mononucleosis caused by Epstein-Barr virus (EBV) have long been heralded as a temporary loss of immune tolerance, potentially not persistent immune sensitization.^1^ Recently this belief has been challenged: studies have demonstrated a noteworthy cohort of patients with persistent hypersensitivity to penicillins after resolution of the acute infection.^2^, ^3^, ^4^ We examined the occurrence and clinical predictors of persistent adulthood penicillin allergy, confirmed by allergy testing, from a cohort of patients reporting penicillin allergy in the setting of reported EBV infection.

We retrospectively examined data from 2066 patients referred for outpatient penicillin allergy testing to an Australian tertiary-care hospital from 2015 to 2023 (Austin Health, Melbourne, Australia). Phenotypic data was collected using a standard template with published definitions, and testing was performed according to previously published protocols.^5^ In brief, skin prick and delayed-intradermal testing (D-IDT) panels included validated penicillin major (benzylpenicilloyl-poly-l-lysine, aka PPL) and minor determinant mixtures, penicillin G, ampicillin, flucloxacillin, amoxicillin, and clavulanic acid, administered at nonirritating concentrations. The results of the D-IDT panel were read at 24 and 48 hours. In patients with negative skin test results, oral provocation was performed at the discretion of the clinician with penicillin VK or amoxicillin. Patients were monitored for 60 minutes after oral challenge, and in most instances, they were prescribed a 3- to 5-day course of the index antibiotic to assess for delayed hypersensitivity. Severe maculopapular exanthem (sMPE) was defined as an extensive cutaneous exanthem covering more than 50% of body surface area and a Registry of Severe Cutaneous Adverse Reaction (RegiSCAR) score of 2-3; drug reaction with eosinophilia and systemic symptoms (DRESS) required a RegiSCAR score of 4-6, as per previously published criteria.^6^

## Results and discussion

Twenty-three patients, 16 female (70%), with a median (interquartile range) age of 38 (33.5-57) years, were identified as having penicillin-associated rash in the setting of reported concurrent EBV infection (Table I). No phenotypes were consistent with anaphylaxis or acute IgE-mediated symptoms. Of the 23 patients, 3 (13%) displayed sMPE, 2 (9%) DRESS, 17 (74%) nonsevere MPE or rash, and 1 (4%) unspecified swelling. Eighteen index events (78%) occurred during childhood or adolescence. Latency between index reaction onset and allergy testing was median (interquartile range) 17 (11-26.5) years. There was no significant difference in the median latency (years) between those positive on testing (6, 26%) compared to those testing negative (17, 74%) (20 vs 11.5 years, *P* = .086).

**Table I:** Characteristics, allergy phenotype, and drug provocation outcomes of 23 study patients

Patient ID	Age at index reaction (years)	Sex	Index drug	Latency (years)	Phenotype	Treatment	Other allergy after index event	Skin testing	Drug provocation testing
1	16	F	Amoxicillin	18	Rash	None		Neg	Neg: Amoxicillin 3d,∗ penicillin 3d
2	31	F	Amoxicillin	15	MPE	None		Neg	Neg: Amoxicillin 3d∗
3	52	M	Amoxicillin	12	Rash	None		Neg	Neg: Amoxicillin S, penicillin S
4	15	F	Penicillin, unspecified	8	Rash	None		Neg	Neg: Amoxicillin S,∗ penicillin 3d
5	20	M	Amoxicillin	14	MPE	Unknown		Neg	Neg: Amoxicillin 3d∗
6	16	M	Penicillin, unspecified	47	Rash	Unknown		Neg	Neg: Amoxicillin S,∗ penicillin S
7	16	F	Penicillin, unspecified	30	Rash	Unknown		Neg	Neg: Amoxicillin S∗
8	30	F	Amoxicillin	35	Rash	Unknown		Neg	Neg: Amoxicillin S∗
9	13	F	Penicillin, unspecified	27	MPE	Unknown		Neg	Neg: Amoxicillin S∗
10	17	F	Amoxicillin	13	MPE	Unknown		Neg	Neg: Amoxicillin S∗
11	Childhood	M	Penicillin, unspecified	>50	Rash	Unknown		Neg	Neg: Penicillin S, amoxicillin∗
12	17	F	Amoxicillin	23	MPE	Unknown		Neg	Neg: Amoxicillin 3d∗
13	29	F	Penicillin, unspecified	19	Unspecified swelling	Unknown		Neg	Neg: Penicillin S, amoxicillin∗
14	20	M	Penicillin, unspecified	8	MPE	None		Neg	Neg: Penicillin 3d, amoxicillin∗
15	20	M	Penicillin, unspecified	20	Rash	Unknown		Neg	Neg: Penicillin S, amoxicillin∗
16	Childhood	F	Penicillin, unspecified	>25	MPE	None		Not done	Neg: Amoxicillin 3d, penicillin 3d
17	15	F	Penicillin, unspecified	38	MPE	None		Not done	Neg: Amoxicillin S∗
18	17	F	Amoxicillin	32	sMPE	Antihistamine, prednisolone	Amoxicillin sMPE	Neg	Pos: Amoxicillin, MPE after second dose; Neg: Penicillin VK 3d
19	18	F	Amoxicillin	16	sMPE	Antihistamine, prednisolone		Pos: D-IDT, ampicillin; amoxicillin†	Pos: Amoxicillin, sMPE, 24 hours after single dose
20	45	F	Penicillin, unspecified	19	MPE	Unknown	Cefalexin, MPE	Neg	Pos: Penicillin, MPE after second dose
21	19	F	Amoxicillin	4	sMPE	Antihistamine	Cefalexin, sMPE	Pos: D-IDT, amoxicillin	Neg: Penicillin 3d; Neg: Cefuroxime S
22	18	M	Co-amoxiclav	3	Probable DRESS,‡ RegiSCAR 5	Antihistamine, prednisolone, hospital		Pos: D-IDT, amoxicillin	Not done
23	19	F	Amoxicillin	7	Probable DRESS,§ RegiSCAR 4	Antihistamine, prednisolone, hospital	Amoxicillin sMPE	Pos: D-IDT, ampicillin, amoxicillin	Not done

*3d,* Single 250 mg dose followed by 3 days of 500 mg twice daily; *Neg,* Negative; *Penicillin,* Phenoxymethylpenicillin; *Pos,* positive; *S,* single dose 250 mg. RegiSCAR scores are as follows: <2 exclude, 2-3 possible, 4-5 probable, ≥6 definite.

∗ Allergy testing patient tolerated treatment course of amoxicillin.

† D-IDT positive in test performed after positive amoxicillin oral provocation challenge.

‡ DRESS defined as sMPE, rash >15 days, fever, eosinophils >1500 cells/μL, lymphadenopathy, atypical lymphocytes, transaminitis.

§ DRESS defined as sMPE, rash >4 weeks, skin desquamation, fever, lymphadenopathy, atypical lymphocytes, transaminitis.

Seventeen patients tolerated a drug provocation challenge (2/17, 12%, direct; 15/17, 88%, after skin testing, 13/17, 76%, amoxicillin; 4/17, 24%, penicillin VK), while 6 patients (26%) were shown to have persistent sensitization (4/23, 17%, positive D-IDT at 24 hours; 3/23, 13%, positive oral challenge) (Table I). All 3 patients with positive oral challenge presented with exanthems within 24 hours of the initial challenge dose. One patient reported a rash the day after a single dose of an amoxicillin direct oral challenge; postchallenge D-IDT was positive. The other 2 patients had negative prechallenge skin test results and developed a rash after 2 doses of penicillin (the first a supervised dose in an outpatient setting, the second the following day at home).

Of the 6 patients with persistent sensitization, 5 (83%) of 6 displayed sMPE or DRESS during their primary index event. Treatment of the index reaction involved antihistamines (5/6, 83%), with the addition of short-course oral prednisolone in 3 instances (50%). Furthermore, 2 patients (33%) had similar exanthems with severe features on reexposure to amoxicillin after the index event, and 2 (33%) experienced a similar reaction after cefalexin treatment in the interim before allergy testing. In contrast, all 17 patients with negative test results had a history of mild exanthems not requiring hospitalization; 7 (41%) of 17 had 3-day oral challenges; 10 (59%) received a single supervised dose of a penicillin; 16 (94%) tolerated amoxicillin in the community after our allergy testing; and 1 (6%) has not required antibiotics since testing.

Our data suggest that although the vast majority of patients reporting penicillin allergy in the setting of EBV infection do not experience a persistent sensitization to penicillin, some patients with a history more consistent with a sMPE may demonstrate reproducible phenotypes on rechallenge distant in time from the EBV infection and index event. This study is novel in that it highlights that this phenomenon can be maintained into adulthood, with potentially initial phenotypic severity as a predictor of persistent sensitization. Although the incidence of penicillin sensitization is limited in the cohort overall (6/23, 26%), it is much greater than adults with persistent penicillin-associated rash developing not in the context of primary EBV (<5%).^7^

Although this study has limitations, including small sample size and absence of serologic confirmation of EBV infection, our findings mirror those of Mori et al,^4^ who in 2019 reported amoxicillin sensitization in 7 of 10 children experiencing delayed exanthems during concomitant co-amoxiclav exposure and serology-proven primary EBV. Similar to our study, they reported severe cutaneous reactions, and separately, patients experiencing a second reaction to β-lactams at a distance from EBV infection, as indicative risk factors for persistent sensitization, and thus allergy. The study of Mori et al, like that in most of the published literature, focuses on pediatric populations, with short latencies between the index event and allergy testing, and thus cannot be assumed to also be true for adults. Importantly, our study demonstrates a novel cohort of adult patients tested for persistent penicillin sensitization after EBV infection, all with >3 years’ latency to testing (median [interquartile range], 17 [11-26.5] years). The current data do support that β-lactam allergy assessment is still required in those patients reporting a penicillin allergy in the setting of EBV where the index reaction was a sMPE.

**Clinical implications**

Delayed penicillin-associated exanthema occurring in the context of childhood EBV infection may not be a transient event. This phenotype may represent a true immune-mediated hypersensitivity that persists into adulthood in those whose index reaction was a sMPE.

## Disclosure statement

Disclosure of potential conflict of interest: The authors declare that they have no relevant conflicts of interest.
